# Remarks on Mr. Bell's Volume on Tumours

**Published:** 1808-09

**Authors:** 


### *To the Editors of the Medical and Physical Journal.*

Gentlemen,

ALTHOUGH Mr. John Bell's splendid quarto volumes have not yet come under the revision of the Reviewers, I think it right that the public, should, in some way or other, be informed that his last volume on tumours, is published, lished, *at present*, in a very incorrect and incomplete manner. So much so, that, with an apology, or without an apology, I never before heard of a work, offered to sale as perfect, abound with so much omission or so many instances of carelessness. This last volume, as the preceding ones, contains numerous plates, which are so connected with the descriptions, that correct references to them are essentially necessary to the perspicuity of the work. But whilst we are reading the description of one plate, we find nothing but references to another. From page 123 to page 174, scarcely a single reference can be found correct. The consequence is, that the reader is interrupted ten or twenty times in every page, by references to plates; and after much time and trouble, he has the mortification to find that some references correspond equally well with half a dozen plates, and others with none at all.

The plates too are numbered after so capricious a manner, that it cannot be known how *many are missing*. The first four plates are not numbered, the fifth is numbered 40. The following plates however are clearly omitted.

1st. Page 112, "the drawing No. 6, of the Ethmoid Bone of a poor woman who perished of this disease" [Polypus.]

2d. Page 114, "the drawing of the little boy, plate 4."

3d. Page 174, "the drawings No. 4 and 5?"

4th. Sketch of Mr. Taylor's, No. 2.

5th. The Sketches of instruments referred to in page 123, and in many parts of the work; but it is impossible to form any idea of the form of the instruments without the sketches, as will appear by one or two quotations, "You will do better to use a knife of the form represented No. 11." Those fashioned like No. 12 and 13, which I have hitherto used." I am conscious that I could manage the form No. 14," &c. &c.

Now, Gentlemen, I apprehend that it is not exactly fair, that imperfect works should be exposed to sale as perfect, at uncommon high prices. I therefore think that the failings in the costly volume above-mentioned, are of that nature as to be a just subject of public complaint. Some notice of the grievance is assuredly called for, if any reparation is to be expected; for the Bells are in the *habit* of leaving their works imperfect. In the second volume of the Anatomy, (chapter the 5th), "on the Structure and Action of the Arteries," is omitted, and has not appeared in any subsequent volume. In the System of Dissections (which work, by the bye, closed very abruptly) a reference is made to a fifth additional plate, and yet only one additional plate was published. I am, &c. B. G.

